# Evaluation of the Impact of Furniture on Communications Performance for Ubiquitous Deployment of Wireless Sensor Networks in Smart Homes

**DOI:** 10.3390/s120506463

**Published:** 2012-05-16

**Authors:** Andrés L. Bleda, Antonio J. Jara, Rafael Maestre, Guadalupe Santa, Antonio F. Gómez Skarmeta

**Affiliations:** 1 Department of Electronics and Home Automation, Furniture and Wood Technology Centre, Yecla 30510, Murcia, Spain; E-Mails: r.maestre@cetem.es (R.M.); g.santa@cetem.es (G.S.); 2 Department of Information and Communications Engineering, University of Murcia, Murcia 30003, Spain; E-Mails: jara@um.es (A.J.J.); skarmeta@um.es (A.F.G.S.)

**Keywords:** Internet of Things, Wireless Sensor Networks, smart spaces, sensing furniture, ambient intelligence

## Abstract

The extensions of the environment with the integration of sensing systems in any space, in conjunction with ubiquitous computing are enabling the so-called Smart Space Sensor Networks. This new generation of networks are offering full connectivity with any object, through the Internet of Things (IoT) and/or the Web, *i.e.*, the Web of Things. These connectivity capabilities are making it feasible to sense the behaviours of people at home and act accordingly. These sensing systems must be integrated within typical elements found at home such as furniture. For that reason, this work considers furniture as an interesting element for the transparent location of sensors. Furniture is a ubiquitous object, *i.e.*, it can be found everywhere at home or the office, and it can integrate and hide the sensors of a network. This work addresses the lack of an exhaustive study of the effect of furniture on signal losses. In addition an easy-to-use tool for estimating the robustness of the communication channel among the sensor nodes and gateways is proposed. Specifically, the losses in a sensor network signal due to the materials found within the communication link are evaluated. Then, this work proposes a software tool that gathers the obtained results and is capable of evaluating the impact of a given set of materials on the communications. This tool also provides a mechanism to optimize the sensor network deployments during the definition of smart spaces. Specifically, it provides information such as: maximum distances between sensor nodes, most suitable type of furniture to integrate sensors, or battery life of sensor nodes. This tool has been validated empirically in the lab, and it is currently being used by several enterprise partners of the Technological Centre of Furniture and Wood in the southeast of Spain.

## 1. Introduction

An extended range of new sensing applications and solutions have emerged in different fields such as rural and forest fire detection [1], monitoring of industrial manufacturing processes [2], transport/logistics [3], and for personal services in areas such as healthcare and Ambient Assisted Living (AAL) [4]. These new advanced applications based on a new generation of sensing and communication capabilities are a consequence of the continuous advances in sensor technologies and wireless communication protocols that allow for greater autonomy of sensor nodes. One of these application areas has focused on Wireless Sensor Networks (WSNs) in the users' environment, such as their home, in order to provide monitoring or assistance in everyday tasks and situations through the denominated Ambient Intelligence (AmI) [5,6]. Specifically, Ambient Intelligence is a multidisciplinary area that combines knowledge from the fields of computer science, electronic technologies, communications and even human behaviour.

The integration of sensors in furniture brings many advantages to AmI systems, compared to other approaches that install sensors independently, for example on the wall. The first advantage is that this allows the definition of ubiquitous can be more easily achieved when sensor nodes are integrated in furniture, since the user will not perceive the existence of these devices, as mentioned by Mark Weiser, the father of the ubiquitous computing, “Hundreds of computers in a room could seem intimidating at first, just as hundreds of volts coursing through wires in the walls did at one time. But like the wires in the walls, these hundreds of computers will come to be invisible to common awareness. People will simply use them unconsciously to accomplish everyday tasks”. Therefore, the integration of the sensors in the furniture is one of the ways to implement an invisible ubiquitous computing environment. The second advantage is that for Ambient Assisted Living solutions, elderly people prefer not to be aware of the existence of the sensors, since this presence is understood as a reminder that they need this support, and it is highly important not to make these deployments highly impacting in people, in order to achieve a higher acceptance. Finally, some pieces of furniture can be in contact with the user, which provides an ideal situation for measuring certain types of variables (e.g., user location, and temperature). This integration of the sensors in typical home objects in conjunction with the capabilities to communicate with remote servers and services provide a high quantity and better quality of data for AmI systems.

This evolution of ubiquitous computing is being extended nowadays with the Internet of Things (IoT). IoT integrates these new capabilities for linking the Internet with everyday sensors and devices, along with ways of communication among people and things, and exploitation of data capture [7]. IoT is considered one of the major communication advances in recent years, which offers the basis for the development of independent cooperative services and applications.

This work is focused on the evaluation of the impact of the furniture on the signal propagation. Since the overall environment includes objects and materials, their distribution will certainly impact the signal communication quality. This work presents the results of an exhaustive set of experiments that evaluate the impact on signal quality of different materials found in furniture, as well as some types of furniture.

In addition to this exhaustive analysis, an estimation tool it has been built, since in most of the cases the performance of a network can only be known after deployment in a particular environment. If some problems are detected they will need to be solved in a second iteration. Therefore, an estimation tool was considered relevant for these situations in order to guide the network designer, and also for the selection of the furniture materials. In this work, we try to solve this problem by proposing a Computer-Aided Design (CAD) software tool for non-expert users. Accurate models require a precise 3-D description of the network and its environment. This description process requires time and knowledge, making it impossible to carry out by final users without technical skills. Our tool aims to simplify this process and only requires a small set of input parameters, while providing good enough results to ensure proper functionality after deployment.

In addition, this provides information about aspects such as the recommendations for sensor location, the required power, battery lifetime, *etc.* Moreover, this tool estimates the signal degradation of different materials, and may be used to guide two different kinds of user: (1) the final user for network design and deployment or (2) furniture designer for materials selection.

The goal of this work is to gather the basic experimental data and knowledge to build this CAD tool. Then, it describes its initial features, experience and results that we have achieved so far. The paper is organized as follows. First, the related work section presents the studies and empirical models that describe the power losses in a wireless network depending on the material. The section also presents the different disciplines involved in the development of an AmI system. Then, the importance of obtaining a completely ubiquitous system with the integration in furniture is highlighted in Section 3. Later, the methodology section describes the methodology to obtain a classification of power losses introduced by most used materials in furniture manufacturing (Section 4). The results are shown and discussed in Section 5. In addition, Section 6 shows a CAD software tool based on the acquired knowledge presented in the previous sections. Finally the discussion section and conclusion section describe the obtained results and the reasons based in analysed data and proposed software.

## 2. Related Work

This work targets the design and deployment of WSN for AmI systems, which requires knowledge from many fields such as computer science, electronics, sensor technologies, and materials science. This paper is focused on two main parts: (1) signal losses and influence of materials, and (2) CAD tools for support on the deployment of a wireless sensor for non-expert users. Consequently, this section is split into four subsections:

- Previous studies of power losses in wireless communication
- Wireless communication technologies.
- Software tools for WSN design and deployment.
- Related AmI projects, since our goal is to support the deployment of WSN for AmI systems.

### 2.1. Power Losses in Wireless Link

There are generic studies based on complex mathematical models, following Maxwell's equations, which allow one to determine physical characteristics and losses introduced by obstacles in the propagation of an electromagnetic wave. However, the complexity of these calculations and the complexity of getting the detailed environment information (materials, distances, *etc.*) make the use of statistical and empirical models the only practical option in many cases. In general terms, there are two main reasons for wireless power losses: “path losses” and “shadowing losses”. The former ones are due to the effects of propagation channel. They are very significant in long distance communications. On the other hand, “shadowing losses” are due to obstacles in the radio link that cause absorption, diffraction, reflection and scattering. In both cases, modelling of the losses based on empirical measurements is a very common approach. Some examples are: free space path loss model [8], Okumura model [9] or Hata model [10] related to path losses, or log-normal model [11] related with shadowing.

Some other empirical studies determine the signal power losses in specific indoor scenarios [12–16], urban environments and outdoors [11,17]. However, in this work we are focused on the signal power losses introduced by specific materials common in furniture manufacturing.

### 2.2. Wireless Communication Technologies

A high number of nodes are usually deployed in an AmI system, which are require to communicate among themselves. These nodes can be classified in three categories: sensors, actuators and drivers (the software part that processes the information coming from sensors, and sends commands to the actuators).

A control network can be considered as a node group with one or more sensors or actuators and some computing capabilities. They communicate over one or more physical media using a standardized protocol. Such networks link the devices in a distributed way, replacing the central controller and resolving connectivity problems of centralized systems, expanding communications possibilities and obtaining information automatically to act on the environment.

There are a lot of technologies, protocols, standards and services to allow communication and integration between devices. Some developments are focused on software modes, for example:

- Jini [18]
- UPnP (Universal Plug and Play) [19].
- OSGi (Open Services Gateway Initiative) [20].

Others are focused on hardware development for control and automation. Currently, among the main technologies for control and automation are:

- X10 [21]
- LonWorks [22,23]
- KNX [24,25]
- Zigbee (IEEE 802.15.4) [26]

We would like to emphasize Zigbee because of its importance in this work. Zigbee is a standard wireless communication developed by the Zigbee Alliance, focused on automation and remote control applications. Zigbee has some features that make it suitable for implementation in Ambient Intelligence environments. Its main advantages are its flexibility, low cost and low power consumption, among others [27]. Zigbee is based on the international standard IEEE 802.15.4, so that interoperability between devices from different manufacturers is guaranteed.

### 2.3. WSN Software Tool

There is a large list of software tools (middleware, operating systems and simulators) for research and design of WSN. This subsection only summarizes different software tools related to wireless sensor networks and highlights the more relevant ones.

Low power microcontrollers, such as MICA/MizaZ [28,29], Tyndall [30], Telos/TelosB [31], and Movital/Jennic [32], are used to provide the increasing functionality of sensor nodes, while meeting the low power consumption requirements in sensor networks. The sensor nodes implement strategies to reduce power consumption at multiple levels, which require the use of specific operating systems such as TinyOS [33], Contiki OS [34], and LiteOS [35]. We are focused mainly on Contiki OS and Movital/Jennic devices.

There are a great number of simulator software tools designed to simulate WSNs [36] such as TOSSIM [37], COOJA [35], and NesCT [38]. Some of them use rather simplified propagation radio models such as COOJA, while others rely on the user to specify a maximum radio range for each node [38]. However, none of these generic software tools consider the signal power losses introduced by specific items like furniture.

The use of CAD tools has emerged due to the complexity of radio signal propagation [36], which is influenced by numerous factors (attenuation, multiple reflections, diffractions…). These tools need the user to input a detailed three-dimensional WSN distribution of nodes for a specific network implementation. Some example are: EDX Signal Pro [39] which supports 3D indoor scenarios; Winprop [40] that uses algorithms like IRT (Intelligent Ray Tracing) in indoor environments; CINDOOR [41] also for enclosed spaces; or Wi-design [42].

Although these tools provide a good level of accuracy, they are far too complex to be used by non-expert users. We aim to develop a tool for furniture designers during the material selection process, as well as non-expert end users who decide to implement a WSN at home. They need a much simpler tool and consequently, we use a much simpler model, even though they may provide only a first order of approximation. This is the reason why we have built a tool on top of empirical results that compare typical furniture materials. With the predicted growth of WSN for AmI and security applications, we think that this tool will be very valuable for non-expert users that want to exploit the new possibilities that this technology provides.

### 2.4. Related Projects

One of the main reasons for deploying WSNs at home is their integration in a full AmI system to improve the end user's everyday life. AmI systems require knowledge from many disciplines such as electronics, sensor networks, computer science, or physical aspects of the materials. Some of the best known projects related to this work are Aura [43] of CMU, Oxygen [44] at MIT, the European Disappearing Computer Initiative [45], and CoolTown [46] from HP. These projects have pioneered the concept of ubiquitous computing based on facilitating a sensors/actuators network infrastructure, and a perceptive intelligent software. These general aspects are similar to our objective presented in this article, since it shares a ubiquitous computing concept based on the sensing/actuation of common objects like furniture and software capable of reasoning and inferring information extracted from sensors, allowing the system to alert of possible anomalous situations and even risks.

Aura is a “Distraction-free Ubiquitous Computing”. Its goal is to increase the user effectiveness by simplifying his interaction with the computer and providing each user with an invisible halo of computing and information services that persists regardless of location. Oxygen enables pervasive, human-centred computing through a combination of specific user and system technologies. It uses a network of computational and hand held devices. However, these projects do not intend to provide an increased functionality to objects around the user. They choose to “watch” using electronic devices such as cameras and microphones that monitor the user's activities.

The Disappearing Computer initiative promotes projects that try to create new functionalities by constructing information artefacts and embedding them into everyday objects (a cup, a shoe, a glass, *etc.*). They do not focus on sensor integration for AmI support.

To the best of our knowledge, there are no projects that exploit furniture as a source of information for monitoring people in AmI systems. However, furniture (chairs, beds, *etc.*) is a great platform for non-intrusive sensing, since it can hide the devices and provide more precise information thanks to its direct contact with the user. Furniture integration overcomes some limitations of other solutions such as clothing. For example, the user needs to change clothes regularly, but the furniture is always in the room. Moreover, furniture provides enough space for integrating any type of sensors, communication circuitry and batteries.

## 3. Ubiquitous Integration of Wireless Sensors in Furniture

The integration of WSN nodes at home furniture, as part of an AmI system, requires the consideration of a number of factors in order to achieve an efficient and reliable system. The integration of sensor nodes in furniture certainly has advantages. For example it increases and simplifies the scalability of a sensor network and its ubiquity. The user does not perceive the existence of the sensors. Therefore, a complete network of sensors can be added without disrupting the room decoration or comfort. In addition, some furniture items can be in direct contact with the user, which can provide high quality measurements. In this case smart textiles could have an important role related to the ubiquity characteristic of the system; their possible integration into the furniture's upholstery could further highlight the ubiquitous feature of a system like this. The smart textiles area has experienced significant advances in recent years. These textiles are able to change their nature in response to the action of different external stimuli in a controlled or predictable way. This is the case of textile sensors. For example, in the case of pressure sensors [47], their electrical resistance varies with pressure. They have been successfully used in sensor carpets and textile keyboards. Another type of sensor whose integration with furniture would not be difficult is the textile temperature sensor [48]; the mentioned smart textiles are highly relevant for their inclusion in furniture because they do not require changes in standard manufacturing processes, and they are invisible to the user, these types of textiles could be integrated under the usual upholstery or even integrated with this. In this area other solutions based on optical fibre sensors for easy integration into textiles have been developed, but this solution is not commercially available yet.

Ubiquitous systems should be non-intrusive and invisible, so that the user never has the feeling of losing privacy at their own home. In this way, the furniture is an essential element that can hide the system to the user. By performing the integration of devices in a WSN in furniture, and deploying it at home, it is feasible to integrate the ambient intelligent system in an environment in a totally ubiquitous way.

In addition, furniture capabilities are increased since they evolve, from passive elements deployed at home with the main goal of providing comfort and welfare state to the user, to active elements providing the ability to interact with the user, and this translates in a greater quantity of applications and features that allow to monitor different aspects within of the home such as detect dangerous anomalies, environment monitoring, checking the daily behaviour of a person that follows a repetitive pattern and infering information about the environment that can be very useful for various applications.

Furniture is the key to achieve this goal, because it allows the integration of sensor nodes in it. Thereby, sensor nodes are hidden to the user and the ubiquitous feature of the system is reached.

## 4. Experimental Methodology

One of the problems found when working with WSN and Radio Frequency (RF) signals are the interactions of the environment with the transmission. Physical objects can exhibit a wide range of behaviours depending on the material composition and signal frequency. An object can be transparent, absorbent or reflective, although most of them exhibit some combination of these behaviours.

First, Section 4.1 describes the materials that are analysed in this study. Later, in Section 4.2, we present the experimental methodology that we have followed in order to evaluate and compare the effects on signal attenuation of a wide set of materials commonly used in furniture. Finally, Section 4.3 is devoted to the description of the experimental methodology for estimating the total impact on signal transmission of complete pieces of furniture.

### 4.1. Selected Materials

Electromagnetic waves are weakened or attenuated when they pass through a material. The amount of power loss depends on the signal frequency and the material. In this study we focus on materials used in furniture manufacturing. According to the Industrial Wood Observatory, the main materials used in furniture manufacturing are wood, metal and plastic [49]. The following summarizes the behaviour of these materials:

- Metal: electrons can move freely in metals, and they are able to oscillate and absorb the energy of a passing wave.
- Wood: the absorption quantity depends on how much water they contain. Old and dry wood is transparent, more or less, but wet and fresh wood presents a high absorption.
- Plastics: this and similar materials usually present a low absorption level, which depends on the signal frequency and the material type.

An important parameter to consider is the water content of materials. Water reduces the power strength of a wireless communication signal [50,51], since the microwaves cause water molecules to shake, capturing some of the wave's energy.

**Figure 1.**
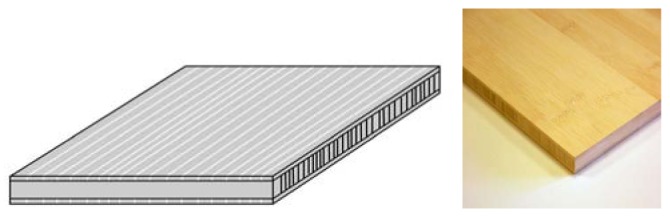
Bamboo images.

**Figure 2.**
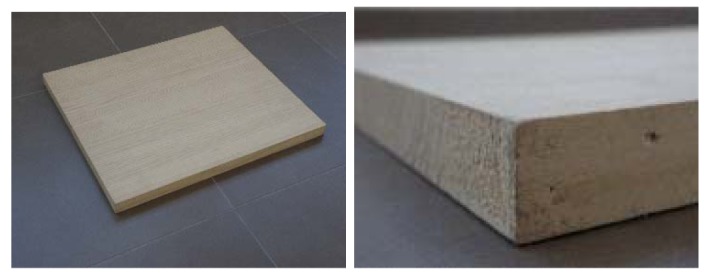
Pine images.

**Figure 3.**
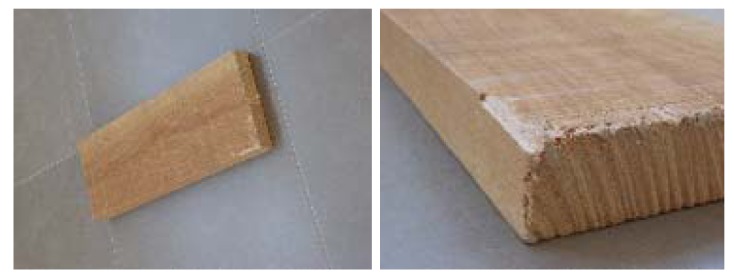
Vaporized beech images.

**Figure 4.**
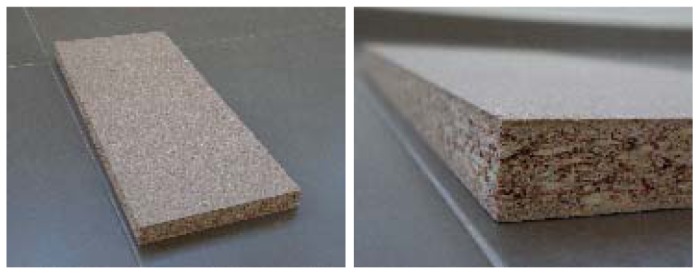
Agglomerated board images.

**Figure 5.**
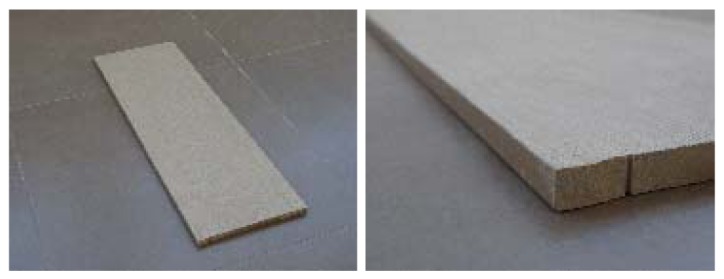
Plastic wood images.

**Figure 6.**
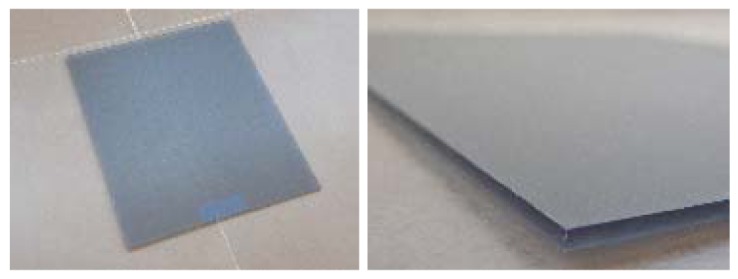
Methacrylate images.

**Figure 7.**
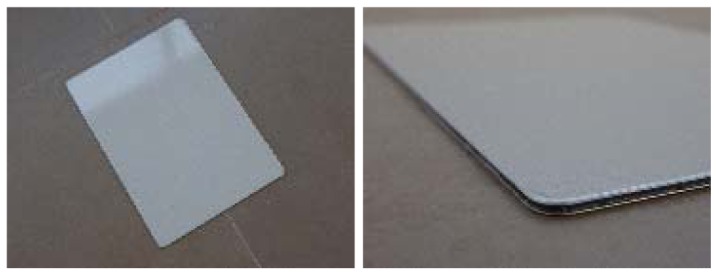
Plastic + Aluminium images.

**Figure 8.**
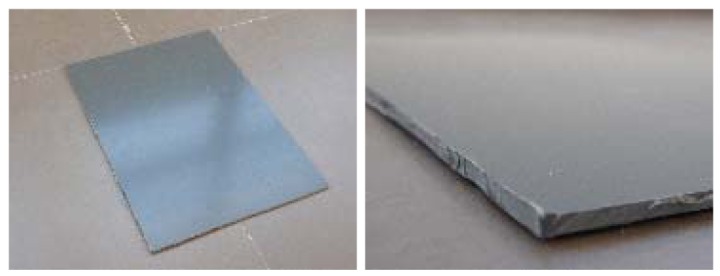
PVC images.

**Figure 9.**
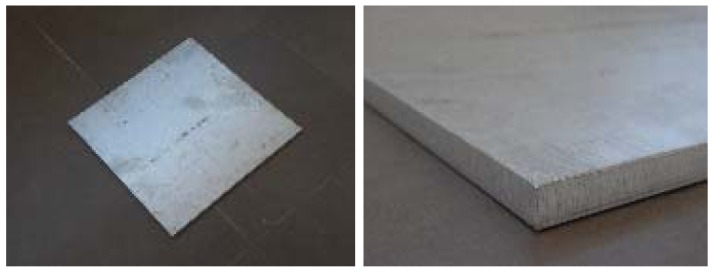
Aluminium images.

**Figure 10.**
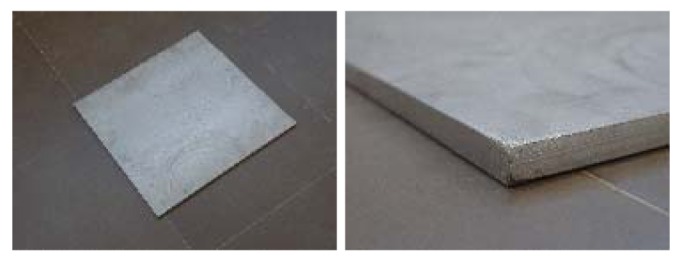
Steel images.

**Figure 11.**
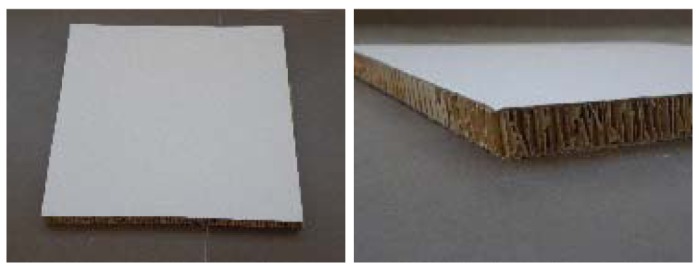
Corrugated cardboard images.

The thickness of any material is a very important factor in relation to attenuation/transmission, but it drastically influences its strength and resistance. In order to carry out a fair comparison across materials, the thickness has to be such that every material can withstand a given weight with the same amount of deflection (bending) [52]. The rest of this section describes the materials considered for the study, and their features:

- *Wood materials*: the most commonly used types of wood for manufacturing of furniture are oak, pine, cherry, chestnut, and beech. To get a representative sample of all of them, the following ones have been selected: pine, beech, agglomerated board, bamboo and plastic wood. The last two are used to make the furniture more attractive to the consumer.
  - **Bamboo** (20.88 mm thick): it is a type of grass with similar characteristics to wood, so its use in furniture manufacturing makes it an excellent material that can help to solve the unlimited exploitation of natural resources. It began to be used for outdoor furniture, but it is now increasingly used in indoor furniture and interior decoration accessories. The drying of bamboo takes longer than wood drying with similar densities. The reason is that bamboo contains hygroscopic materials (substances that absorb humidity easily) and can contain from 100% to 150% of humidity; levels depending on the time of harvest, the growing region and the type of bamboo.
    According to the technical characteristics of the bamboo samples (Figure 1) used in the experiment, the humidity quantity content ranges between [53]:
    10% at 20 °C and 65% relative humidity
    8% at 20 °C and 50% relative humidity
  - **Pine** (25.5 mm thick, Figure 2): it is nowadays one of the most used woods on the market due to its affordable price, quality and hardness. It is mainly used in furniture manufacturing, flooring and building finishes, as well as other purposes in carpentry and construction.
    For the experiment, glued “insignis” pine boards have been used, which according to their technical characteristics present an 8 ± 2% humidity [54].
  - **Vaporized beech** (35.51 mm thick, Figure 3): beech wood is durable and resistant to abrasion. Its good physical properties and resilience to shocks make beech wood preferred for use in areas subject to wear and friction. Beech is available vaporized or not, the vaporization enhances the malleable characteristics of wood and brings out the natural colour of wood with a reddish glow. It is especially used in furniture manufacturing and in creating turned items. Among its best features are a good price and a finish of great aesthetic level. It contains 9 ± 1% humidity.
  - **Agglomerated board** (16.33 mm thick, Figure 4): agglomerate is derived from wood and is manufactured from wood wastes, for example wood shavings, untapped splinters, *etc.* This material is easy to cut and tough enough so it is widely used in manufacture of all types of furniture. The agglomerate used in this evaluation is a veneered agglomerate with has faces coated with a natural wood veneer, and contains 8 ± 3% humidity [55].
  - **Plastic wood** (9.9 mm thick, Figure 5): it is a material made from the extrusion of a thermoplastic polymer in pellet form with wood fibres or particles which come from forest industry, mainly sawdust and virgin or recycled thermoplastic polymers. Recycled plastic is more resistant than wood, is sterile, imperishable, a good insulator, insensitive to water, humidity, chemical factors, ultraviolet (UV) rays and living organisms such as bacteria or insects. In addition, it is easy to clean and disinfect, and it has zero humidity content.
- *Plastic materials*: plastic has gradually gained acceptance in the decoration sector. It is used as a material for the manufacture of low-cost and casual accessories; but it is also a material associated with furniture and avant-garde design, because it is versatile, lightweight, colourful, and not very expensive.
  Furniture made of plastic are typically very flexible and has a truly dynamic behaviour, plus endless possibilities of colours and finishes, which are characteristics and details that cannot be achieved in the case of wood. The plastic elements most commonly used in furniture are polycarbonate, polyethylene and methacrylate, which can be perfectly transformed and modelled in the transformation process resulting in fabulous design pieces. The plastic samples used for the evaluation are:
  - **Methacrylate** (8.12 mm thick, Figure 6): it is a material that has been introduced gradually in interior design and decoration. From a functional point of view it has interesting properties such as strength, which makes it suitable for indoor and outdoor aplications, and from the decorative point of view, the variety of finishes, gloss, matt, plenty of options in colours, makes methacrylate a very interesting option. It contains 0.1–1% humidity [56].
  - **Plastic + Aluminium** (2 mm thick) (Figure 7): the “Dibond” material is a panel composed of two layers of aluminium and a polyethylene core. This group of materials presents an extraordinary flatness and also excellent mechanical properties. The selection of this material is due to the combination of individual characteristics. Aluminium sheet provides the necessary strength and rigidity, and the polyethylene core provides the necessary flexural properties for the material to absorb the deformations due to the weight placed on the final product without breaking.
  - **PVC** (3.3 mm thick, Figure 8): it is the most practical material for kitchens due to its strength and easy cleaning. It is a plastic material of the highest quality and durability and with better characteristics than traditional wood and agglomerated wood. It contains 0.1–1% humidity [56].
- *Metallic materials*: metals are usually reserved for places such as the bathroom or kitchen, but are increasingly being integrated in all rooms of the house. Aluminium and steel are the most widely used metals:
  - **Aluminium** (10.39 mm thick, Figure 9): it is a light material and as strong as steel. Its extended use in many applications, lacquered or natural, has increased the options for manufacturing structures in modern and simple furniture and with an up-to-date style.
  - **Steel** (8.17 mm thick, Figure 10): it is one of the most in-demand furniture materials, since it ensures greater durability, lower expenditure on maintenance products and less cleaning time. In addition, it offers a very powerful style, beauty and perfect functionality. It is used for the design of furniture for kitchen, bedroom, garden and office objects.
- *Cardboard* (16.2 mm thick, Figure 11): Cardboard furniture is ecological, economical and original traditional wooden furniture. Furniture manufacturers use this material to reduce costs and weight of the final products. It is used to make tables, cabinets, bed heads, and doors. Two types of cardboard furniture are found: corrugated and the reinforced.
  - **Corrugated cardboard** is a structure formed by a central nerve of corrugated paper, externally reinforced with two layers of paper. It is a light material, whose resistance is based on the vertical joint and its three sheets of paper. For the most resistance, wave board is to work vertically.
  - **Reinforced cardboard** is suitable for obtaining a certain type of furniture or other objects to contain a plurality of rods or wire mesh between two layers of cardboard.
    For this experiment, the corrugated cardboard with honeycomb and hexagonal structures that gives extreme rigidity suitable for the manufacture of all types of furniture has been used.

### 4.2. Methodology to Compare the Attenuation of Different Materials

The experiments are based on the value of Link Quality Index (LQI). The LQI concept is similar to Received Signal Strength Indication (RSSI), since it is an estimate of the quality of the communication link. The following formulas show how to calculate it:

$$\begin{array}{c} \text{LQI}=(47-\text{MED})\times 6\\ \text{If}(\text{LQI}<0)➔\text{LQI}=0\\ \text{If}(\text{LQI}>255)➔\text{LQI}=255 \end{array}$$

where MED = Number of gain stages required to receive that packet. Currently there are 47 gain stages and each adds 2 dB to receiver sensitivity. A LQI value lower than 30 is considered a bad link.

**Figure 12.**
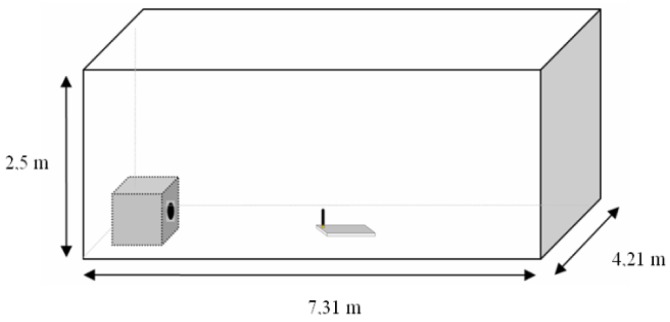
Testing environment dimensions.

**Figure 13.**
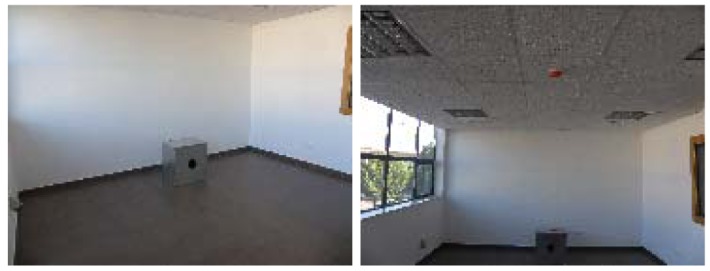
Testing environment images.

The first set of experiments is focus on measuring LQI results of different materials under the same testing conditions. The tests were performed in a closed room with dimensions 4.21 × 7.31 × 3 meters (Figures 12 and 13).

**Figure 14.**
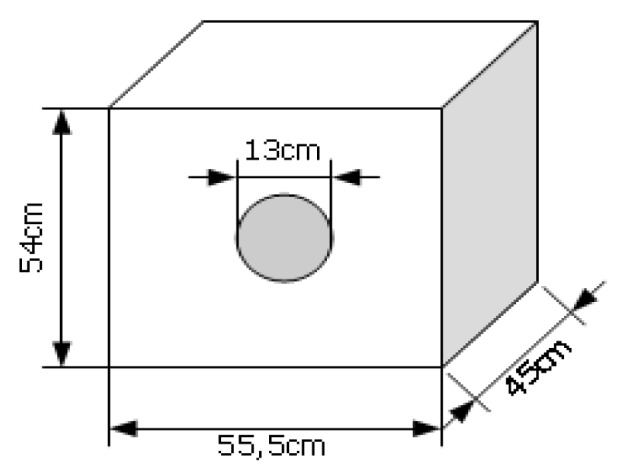
Metal box dimensions.

**Figure 15.**
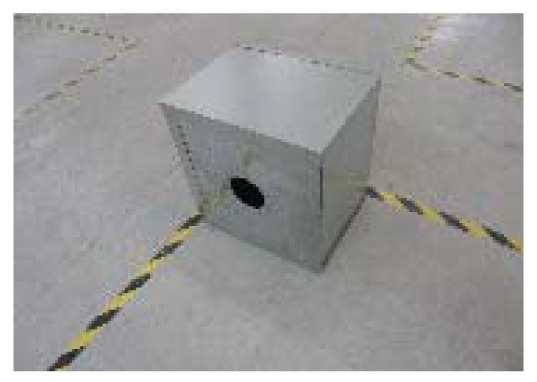
Metal box image.

**Figure 16.**
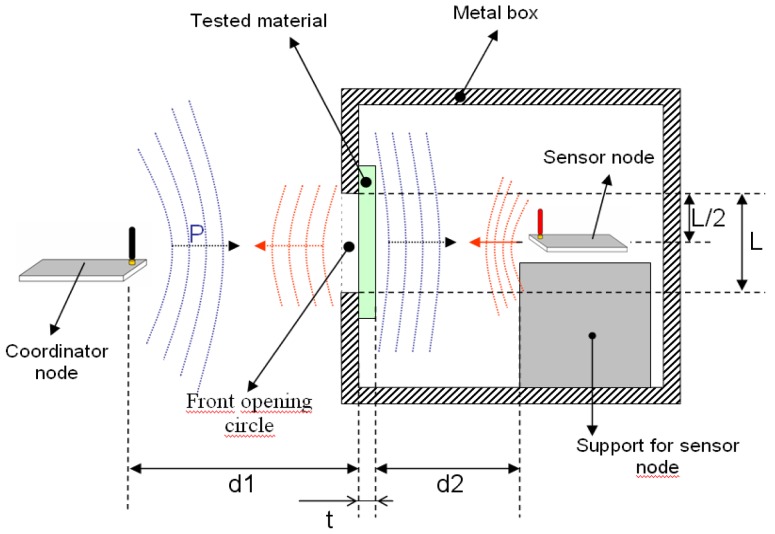
Testbed diagram.

**Figure 17.**
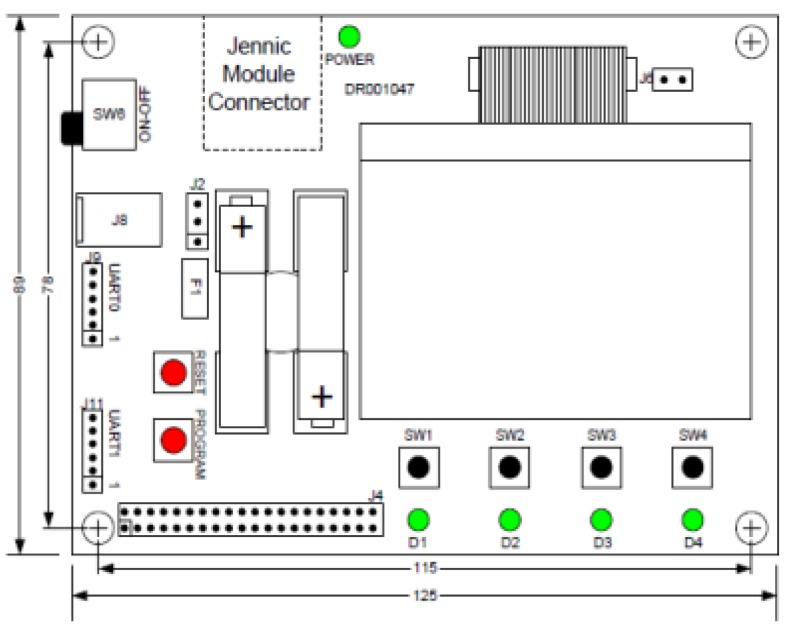
Zigbee coordinator.

**Figure 18.**
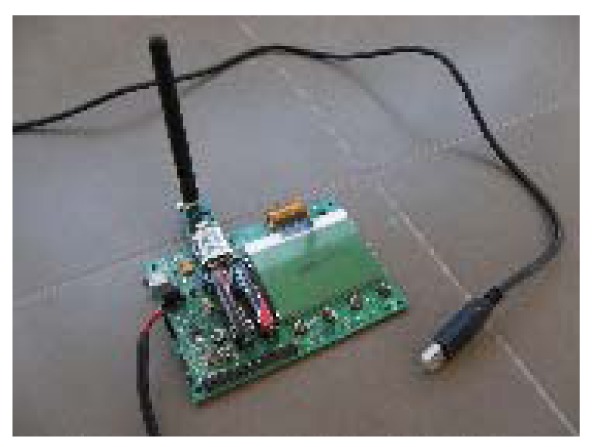
Zigbee coordinator.

**Figure 19.**
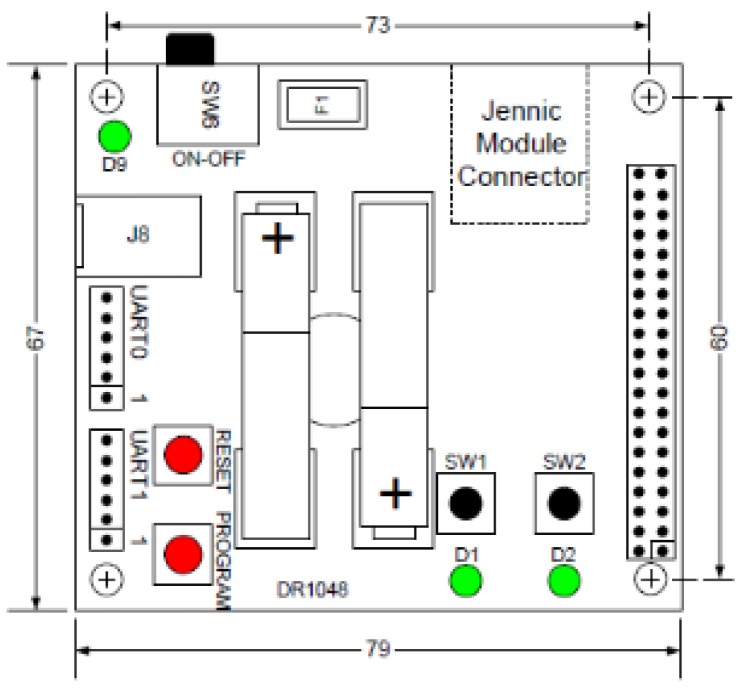
Zigbee sensor board.

**Figure 20.**
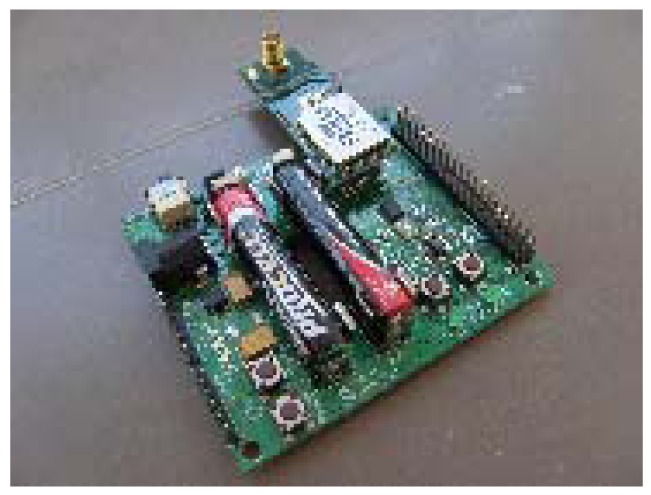
Zigbee sensor board.

The following items have been necessary to perform the tests:

- Metal box with a circle-shaped front opening: the purpose of the metal box is to strongly reduce the wave's transmission through its walls. The box has a front opening circle so that the main contribution to the received signal will be the straight line through the whole between transmitter and receiver. Reflections on external objects will contribute with much smaller power. If a particular material is placed to block the front opening, LQI is mainly affected by the material properties. Even if some reflected signals contribute to the received signal, they will be almost the same for different materials if exactly the same environmental conditions are maintained. In this case, a relative comparison of LQI values is still valid. The box dimensions and a picture are shown below (Figures 14 and 15):

The following image (Figure 16) shows the inside diagram of the box, and it also shows materials position and distances:

- Zigbee coordinator (Figures 17 and 18): It is a controller board acting as coordinator. It is provided with a “JN-5148 Evaluation kit”. This board contains a 128 × 64 pixel LCD screen, four configurable LED indicators, four configurable push buttons, a temperature sensor, a humidity sensor, a light sensor, serial EEPROM, expansion ports to add more sensors and an UART interface communication to communicate and program the board. This UART is used to transfer data to the computer.
- Zigbee Sensor Board (Figures 19 and 20): this sensor board is also provided with a “JN-5148 Evaluation kit”. This board contains two configurable indicators LEDS, two configurable push buttons, a temperature sensor, a humidity sensor, a light sensor, a serial EEPROM memory, expansion ports to add more sensors and an UART communication interface to communicate and program the board.
- USB peripheral cable: the USB is used to transfer information about LQI value received by controller board to the computer, where data is stored for later analysis
- PC for data storage: USB port receives the LQI value and stores it for later analysis.
- Tested materials: the tests are performed with different materials that are commonly used in furniture manufacturing. These materials include the wood, plastic and metal families. When electromagnetic radiation passes through a material, it is usually absorbed and attenuated. The amount of power lost depends on the frequency and the characteristics of the material.

The experimental methodology is made up of the following steps:

1. Coordinator and sensor node programming:
  - The software is based on Jennic Application “Packet Error Rate Testing” (JN-AN 1006 [57]). We made small changes in the coordinator program because we only want to consider the LQI value, which is returned via the serial port.
  - Coordinator and sensor nodes are programmed as shown in the JN-AN 1006 application note.
2. The sensor node is placed inside the metal box (always in the same location/distances from box walls and bottom).
3. The material to be tested is placed so that it completely covers the opening of the metal box, on the inner side.
4. The coordinator node is placed at 5 meters away from the metal box.
5. The coordinator node is connected to the PC via USB.
6. HyperTerminal application is run on the PC to connect with the coordinator node:
  - Select port to connect coordinator.
  - Configure port parameters:
    - 38,400 bits per second.
    - 8 data bits.
    - No parity.
    - Stop bits: 1.
    - Flow control: None.
7. In the coordinator node the following options are selected:
  - Channel: 11.
  - Power Mode: Low.
  - Power Level: 24.
  - Retries: 0.
  - Leds: Disabled.
8. After selecting “Done” the experiments begins and the LQI values are continuously dumped to a text file for later analysis. We obtain 1,000 samples of LQI value for each material.

### 4.3. Methodology to Measure the Electromagnetic Influence of Complete Pieces of Furniture

**Figure 21.**
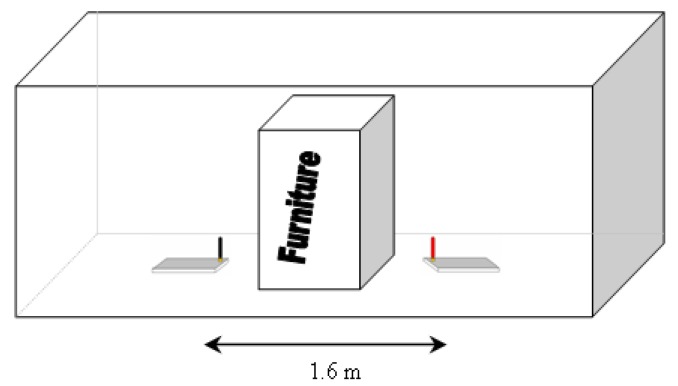
Distance between sensor and coordinator nodes.

The second part of test development, LQI parameter value measurements are made assuming that a particular type of furniture is the unique obstacle in communication link. To do this, only furniture is placed between the sensor node and the coordinator node. These measurements are made by placing furniture in direct communications link between sensor and coordinator node (Figure 21).

The furniture models considered in this part are:

- Recliner armchair with metal structure.
- Room table with pine wood structure.
- Wardrobe with beech wood structure.
- Bathroom cabinet with plastic wood structure.

The distance between sensor and coordinator node is 1.6 m.

## 5. Results

The first step in our testing procedure consisted in comparing the LQI value with and without the metal box. Its goal is to validate our assumption that the straight line is the main contributor to the received signal. In the second part of the experiments, different materials cover the front opening of the box and a comparison between materials within the same family is carried out. Finally, we present a comparison between all selected materials.

### 5.1. Behaviour of Metal Box

**Figure 22.**
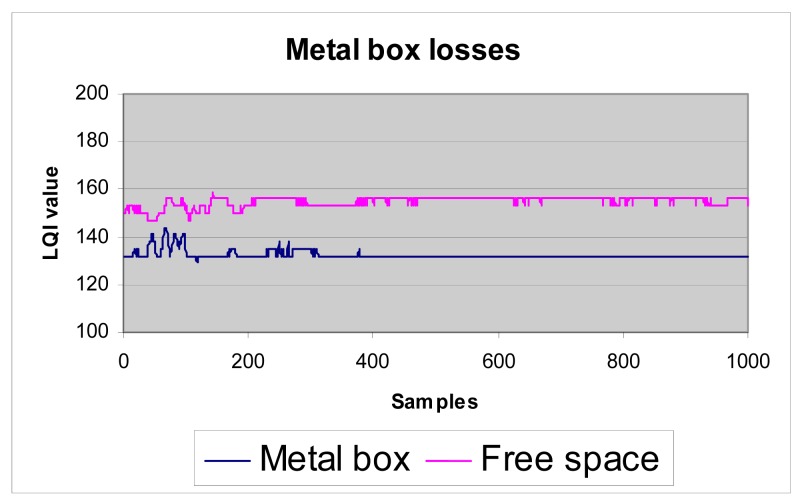
Measures with metal box and free space.

In order to get an estimate of the metal box losses, we obtain LQI value measurements without metal box (in free space) first, and later using the box. Shown here (Figure 22) is a comparative graph from an experiment with a 5-meter distance between sensor and coordinator nodes:

The received signal strength without the metal box is clearly above the signal strength using the box; this result was predictable and confirms that the testbed fulfils its purpose. The losses represent about 20 units of lower LQI value.

### 5.2. Results for Different Materials

This section describes a comparison between the obtained LQI values for different materials. First, a comparison among materials within the same family has been carried out. Then a comparison among all selected materials is presented.

A realistic comparison among different materials must consider their relative thickness, so that all of them provide the same amount of strength. In other words, weaker materials must be thicker, and stronger materials must be thinner. The actual thicknesses have been carefully chosen to provide the same amount of strength across materials.

**Figure 23.**
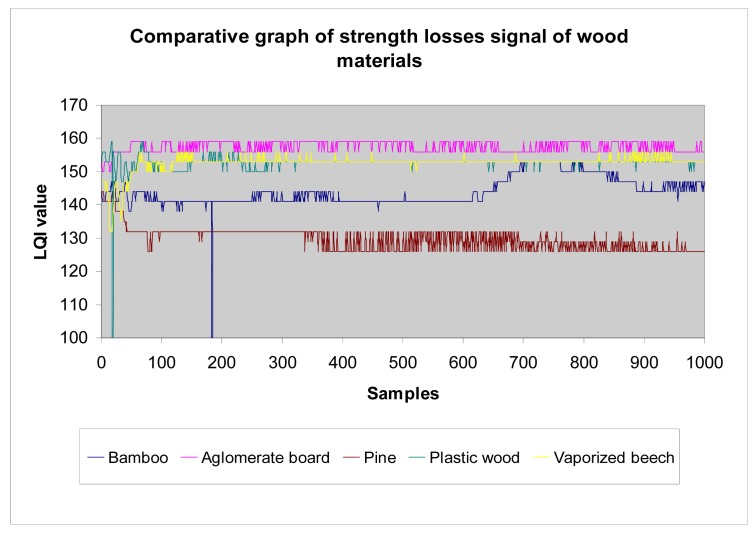
Measurements with wood materials.

**Figure 24.**
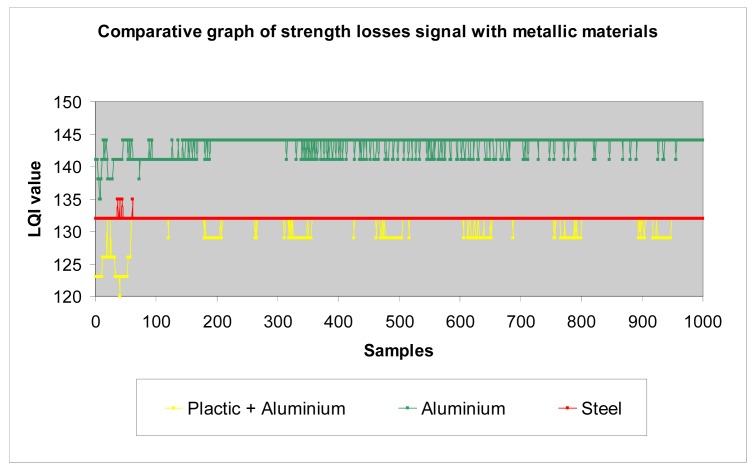
Measurements with metallic materials.

**Figure 25.**
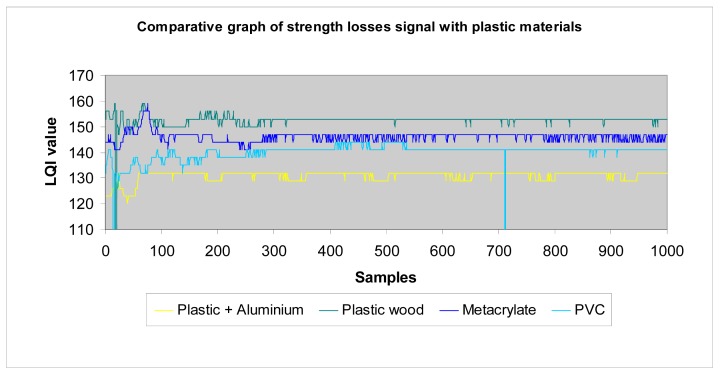
Measurements with plastic materials.

- *Wood*: Different wood structures have been considered in the tests. The following graphs show the results that allow making a comparison of losses introduced by different wood types to the transmission of waves. The higher the LQI value, the lower the losses introduced by the material. Table 1 shows the thickness considered for each one of the different wood structures, in function of their density.
  The analysed data (Figure 23) shows that agglomerate board, plastic wood, and vaporized beech have very similar level of losses, and pine has the greatest amount of losses, followed by bamboo. We make the following classification (Table 2):
- *Metallic materials*: similarly to previous paragraph, we make a comparison between different metals considered in the tests (aluminium, steel and a plastic + aluminium structure) (Figure 24).
  In general terms, as we show later in the section, metals have higher losses than the rest of materials. This is an expected result and confirms the testbed is well-designed.
- *Plastic materials*: the same approach has been carried out for plastic materials (Table 3):
  The results are shown in Figure 25. One can easily observe that the plastic + aluminium structure has higher losses than the rest. This was expected because it contains two layers of metallic materials. The other plastics materials have similar behaviour among them. Their losses are in the same rank, but it is clear that plastic wood presents the best performance and has the lowest losses, even with a triple thickness. We make the following classification (Table 4).
- *All materials*: the following graph (Figure 25) summarizes the results of tests performed with all materials.

The graph shows that LQI has the greatest value when no material is covering the opening metal box, as would be expected. It also confirms that metallic materials have a greater amount of losses, this result was also predictable. For some materials the LQI values are very close and even overlap in the graphs (*i.e.*, cardboard, pine and plastic+aluminium), so we need a second criterion in order to perform a fair comparison. In the previous experiments, different materials have been chosen to provide the same level of strength, which implies a different thickness for each material. However, there are other reasons besides strength, like aesthetics, that can determine the thickness of a material in a piece of furniture. In these situations its thickness is usually a fixed value. Consequently, if two materials have a very close LQI value, a thicker sample means it introduces lower losses. After analysing the data, we establish the following classification of materials (Table 5), starting with those that introduce lower losses and ending with materials with greater losses:

### 5.3. Results for Different Pieces of Furniture

As mentioned previously, these measurements are made by placing different furniture pieces between the coordinator and sensor nodes. Four different types of furniture were selected with four different structural materials:

- Recliner armchair with metal structure.
- Bedside table with pine wood structure.
- Wardrobe with beech wood structure.
- Bathroom cabinet with plastic wood structure.

**Figure 27.**
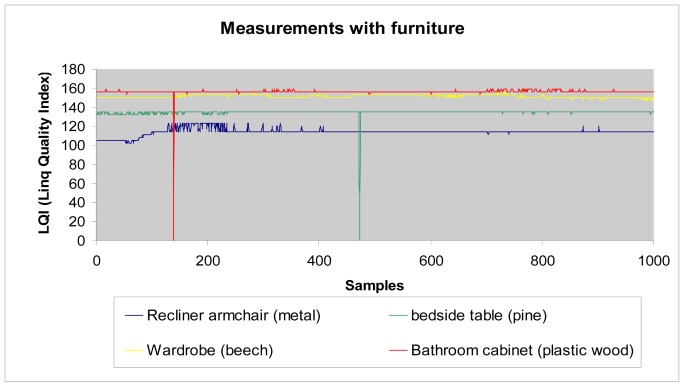
Measurements with different furniture.

As in previous section, 1,000 consecutive measurements of LQI value are taken and shown in the following graph (Figure 27). The highlight of these data is the LQI value when the obstacle between sensor and coordinator node is the recliner armchair, made of metal. For this furniture, the LQI value is smaller than for the other furniture, as expected from the results obtained in the previous section. On the other hand, the plastic wood structure introduces lower losses than pine or beech. Finally, the wardrobe (beech wood) has lower losses than the bedside table (pine). These results show that we can establish a direct relation between measurements obtained with materials and complete pieces of furniture.

### 5.4. Discussion of Results

After performing experiments that have characterized the losses in the communication signal strength caused by different materials commonly found in furniture structure, we can conclude that:

- In general terms, there are wood materials, like agglomerate board or vaporized beech with lower losses than other considered materials; although a few plastic materials introduce a signal loss within the same range as some wood materials.
- As expected, furniture with metallic structures display high communications signal strength losses, so that their integration in environments where one might want to deploy a sensor network will damage the communication link. In this case, there will be a worse link than with another furniture structures.
- Another aspect to consider is the battery life of sensor nodes, which arises from the previous study. If the introduced losses in the signal are high enough, there will be a greater amount of packets lost in the transmission, having to forward the same information packets many times, and as a result this will increase the energy consumption, and the battery life of nodes will last less.

## 6. Software Tool Description

The purpose of the above-described study was to characterize the losses introduced by different types of materials commonly used in furniture, as well as different pieces of furniture. Manufacturers can check the results of this study as a guide to choose which materials can perform best in a WSN environment. This information can also be used to design and evaluate the performance of a new deployment. However, this type of design decisions will be much easier to make if all the information is wrapped into a software tool that automates the whole process and produces recommendations depending on user-defined parameters.

This section describes the developed tool. It also analyses its full potential and proposes additional capabilities that the tool could provide and how to implement them.

There are clearly two potential uses for this tool:

- Selection of materials for furniture design and integration of sensor nodes.
- Design and deployment of a sensor network at home.

**Figure 28.**
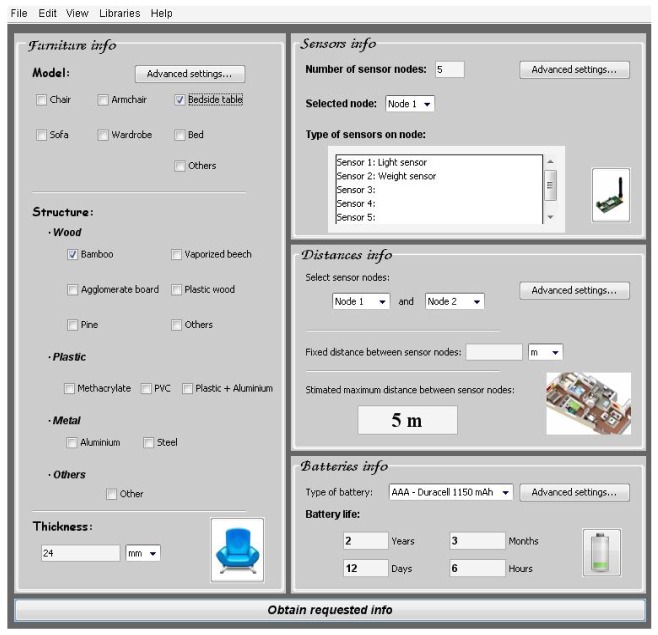
Main software tool user interface.

This tool has a main and a summary window (Figure 28) which allows the furniture manufacturers and sensor network designers/engineers to make quick one-click selections and access to the advanced settings menus. The next image shows the main screen of the software tool GUI:

The main window is divided into four main window sections, and it is possible to access to advanced settings in each of them. The four sections are:

- Furniture information: type of furniture, main material in the furniture structure's and thickness of the furniture structure.
- Sensor information: total number of sensor nodes, sensor types per node.
- Distance information: the real deployment of a WSN at home depends on the real available space and the home distribution. This window section allows introducing some information like distance between sensor nodes, floor plan available or number of walls between sensor nodes. Depending on the advanced settings, the tool can otherwise estimate the maximum distance between a pair of selected sensors (as shown in Figure 28).
- Battery information: the tool can estimate the battery life of each sensor node. This practical and valuable information is showed in this window section.

In the following subsections practical and potential uses for this tool are explained.

### 6.1. Furniture Design and Integration of Sensor Nodes

**Figure 29.**
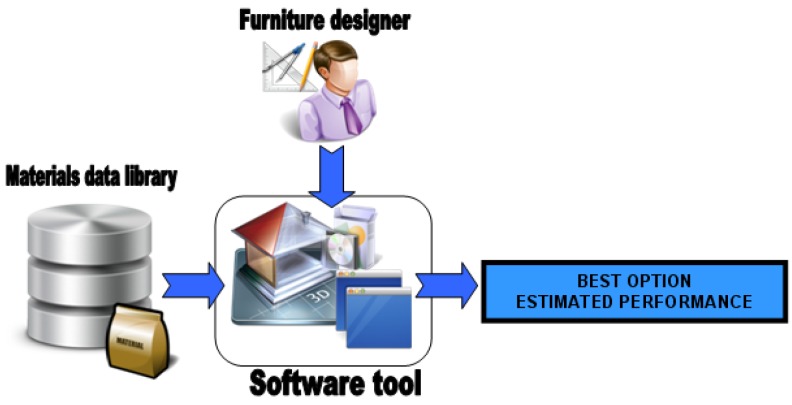
Software tool concept in furniture factory.

**Figure 30.**
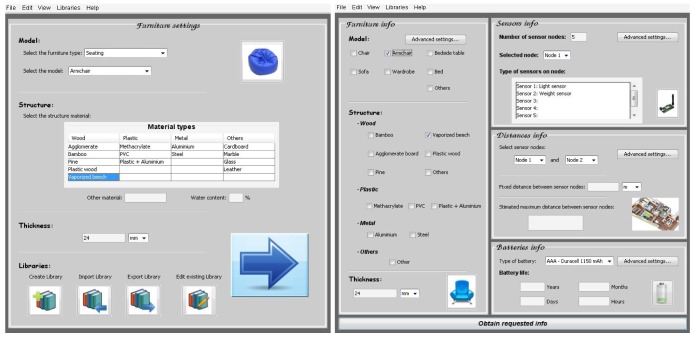
Case A: Introducing furniture information in the software tool.

**Figure 31.**
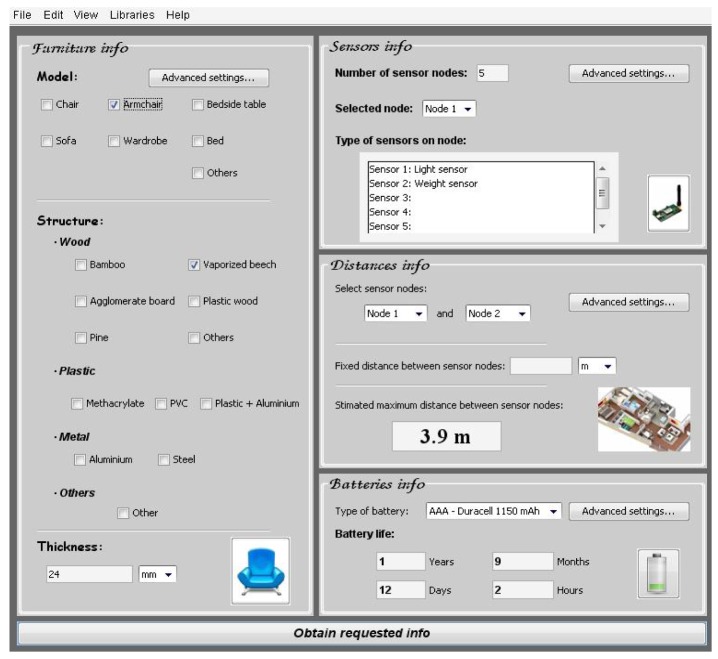
Case A: Information obtained from the software tool.

**Figure 32.**
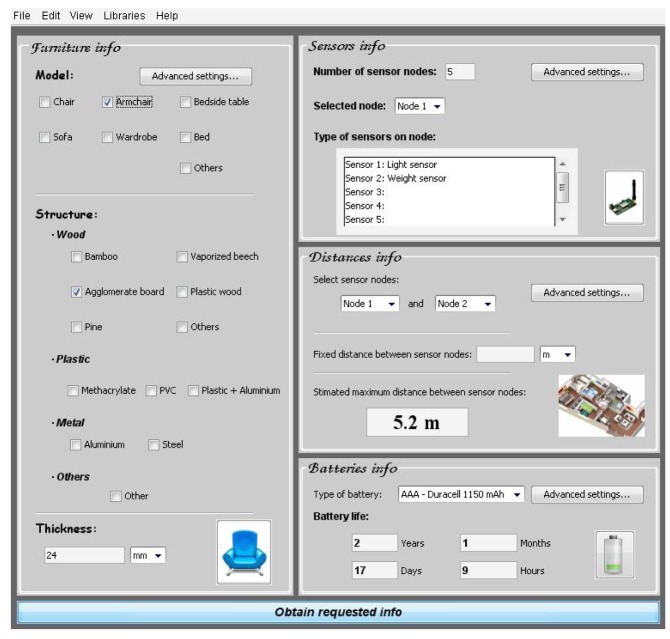
Case B: Information obtained from the software tool.

All materials that can be used in furniture manufacturing have been characterized in order to measure the losses they introduced in a communication link (as explained in Section 5.2). This information makes up a library with the results of the characterization of losses introduced by each material. The software tool allows the selection of some input options like type of furniture, preferred materials and their thickness. Finally the software tool estimates the performance of the best possible combinations among the ones that meet the selected input options. The following figure shows a scheme (Figure 29) illustrating this potential use of the software tool:

The software tool provides an easy way for the furniture designer to make the selection of proper materials for furniture manufacturing, considering that a WSN will be integrated in it. For example, when a furniture company integrates sensor nodes in its products and the company can manufacture a specific type of furniture (chair, bed, armchair, *etc.*) with different materials (bamboo, methacrylate, beech, *etc.*), the designer can compare and evaluate the alternatives. Following, a practical use is described:

A furniture company has to deploy a WSN in their furniture into a specific house, and they have the possibility to manufacture an armchair with vaporized beech or agglomerate board with a specific thickness. The software tool helps to select the best choice:

**Case A**: Armchair of vaporized beech and 24 mm thickness. Light and weight sensor integrated, and with AAA −1,150 mAh batteries type (Figure 30).
  After introducing the furniture description about the structure material, the type of sensors and the batteries information, the software tool shows the maximum distance between sensor nodes and battery life (Figure 31).
**Case B**: Armchair of agglomerate board and 24 mm thickness. Light and weight sensor integrated and AAA −1,150 mAh batteries type (Figure 32).

In this practical example, the company can make an appropriate selection of furniture material because they know more information about the maximum distance between sensor nodes (3.9 meters with vaporized beech and 5.2 meters with agglomerate board) or the battery life (longer with agglomerate board). Taking into account this information and others, like the structure material price, the company can make a proper selection.

### 6.2. Design and Deployment of Sensor Network

**Figure 33.**
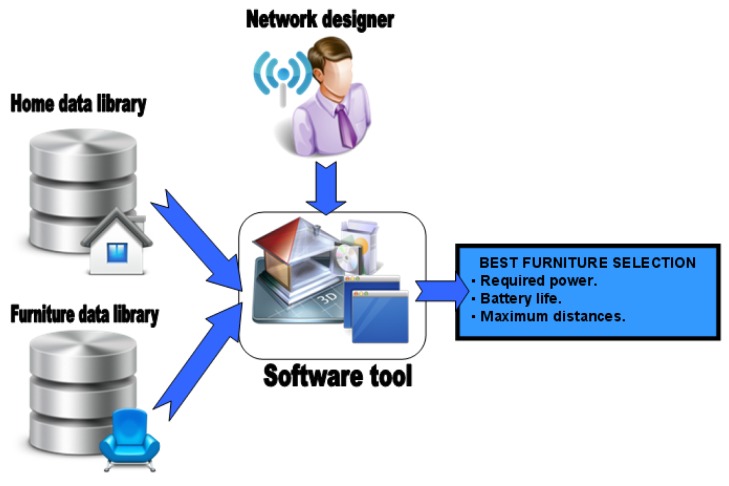
Software tool concept in furniture sensor network deployment.

This tool is also used to deploy the sensor network in home-furniture items. It will provide an estimate of the performance in required power, battery life, distances between sensor nodes, *etc.* In this case, furniture is previously characterized after fabrication either in the factory or on-site. Based on these results a library will be build. Then, with the furniture library and information about home dimensions, the network designer could introduce other data like home floor plan, sensor types and location, furniture distribution in rooms or furniture options and the software tool will make the best furniture selection for that specific case. The next figure (Figure 33) shows a diagram about this use:

#### 6.2.1. Furniture Characterization

**Figure 34.**
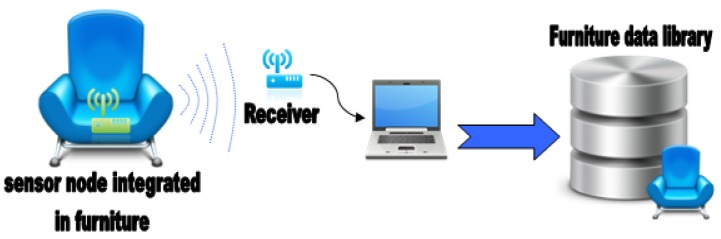
Data acquisition for furniture data library generation.

To develop the furniture data library, there are two main options. On the one hand, the main manufacturer can characterize their furniture in their own factory, integrating a sensor node in the selected furniture and placing a receiver node at a specific distance, and then the obtained values will be transferred to a personal computer to develop the furniture library. On the other hand, it is possible to characterize furniture losses on-site, integrating sensor node in pre-placed furniture at home and obtaining the information similarly than in factory option (Figure 34). In this case the obtained results will be better adapted to the specific home.

**Figure 35.**
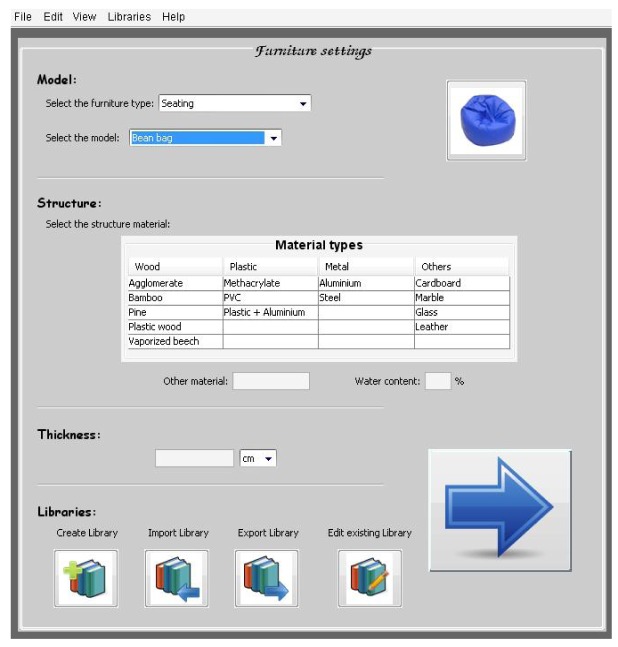
Screenshot of furniture settings information.

The software tool includes these options (Figure 35) in the advanced settings of the furniture section where many options can be selected and it is possible to create, import, export or save furniture libraries. During the creation of the library, the information regarding the characterization is requested.

#### 6.2.2. Home Characterization

**Figure 36.**
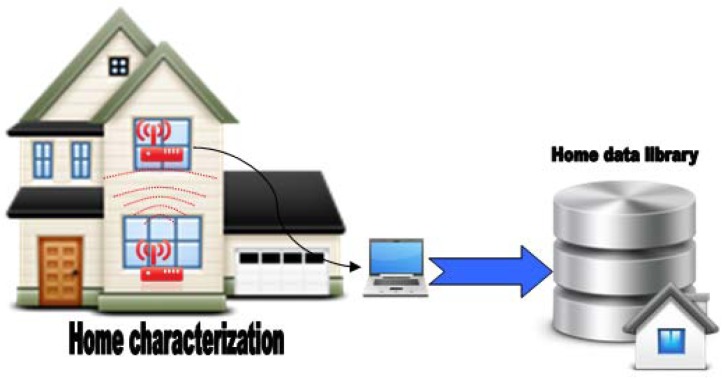
Data acquisition for home data library generation.

In a generic house we can find different rooms with distinct characteristics. Dimensions can change from one room to another, objects placed in rooms are not the same, and all changes can affect a wireless sensor communication link. For all of these reasons it is advisable to characterize the house and obtain as much information as possible. Distributing one transmitter and one receiver for every pair of adjacent rooms and later analysing the obtained data (Figure 36) will be enough to characterize the house.

**Figure 37.**
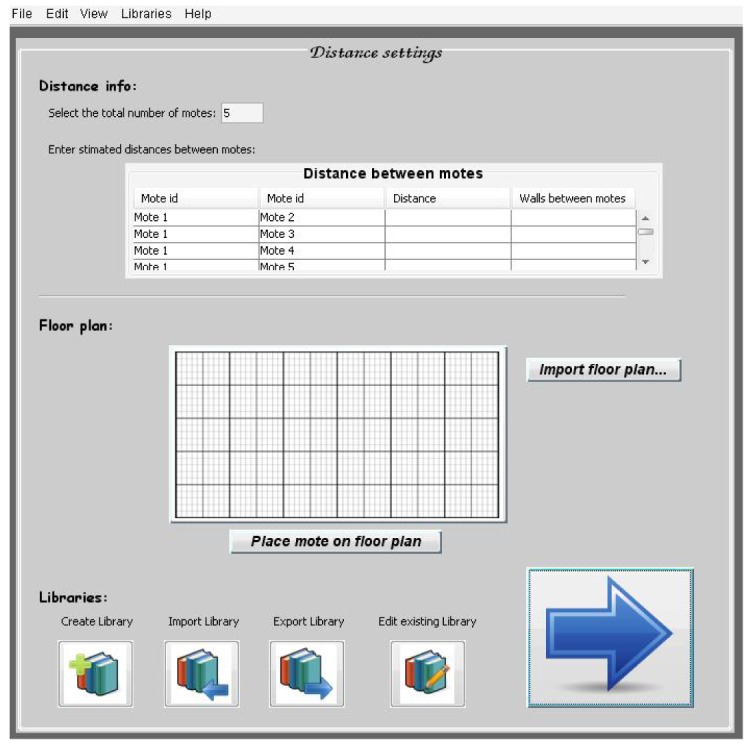
Screenshot of home settings characterization.

In the advanced settings of distances section, the software tool user can select aspects related with this information. The user can import, export, create or modify a library and also include information about distance between sensor nodes, number of walls between motes or import a real floor plan (Figure 37).

#### 6.2.3. Information Capture Window for Sensors

**Figure 38.**
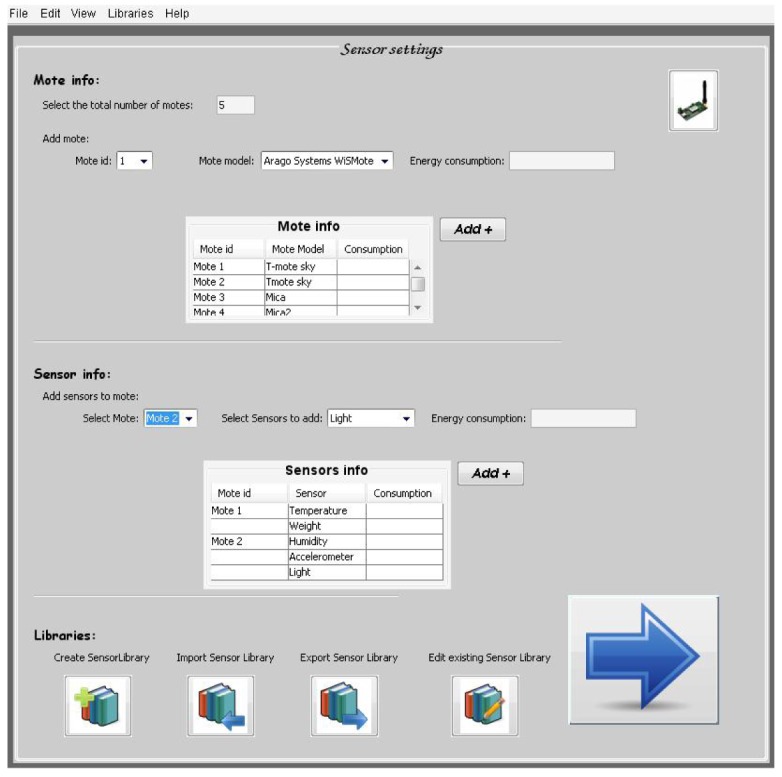
Screenshot of sensor settings characterization.

There is other sensor information section where the software tool user introduces specific information about the integrated sensors on the mote, the mote model or the mote energy consumption (Figure 38). This kind of information is used by the software tool and leads to a complete tool very useful for a wireless sensor network deployment at home.

## 7. Conclusions and Future Work

In the Internet of Things and ubiquitous computing solutions such as Ambient Assisted Living deployments, it is really important to increase the ubiquitous characteristic of the system and furniture can play a key role in this. On the one hand furniture allows a seamless integration of sensor nodes in it. If the user does not perceive the existence of these devices, the system could be totally ubiquitous without really changing the user's environment. On the other hand, some sensors integrated in furniture would be in direct contact with the user allowing measurements such as body temperature, or humidity (for example, urinary incontinence for the mentioned Ambient Assisted Living scenario). Furthermore, it could also obtain measures of other variables such as furniture occupancy, room temperature and luminosity, opening and closing doors.

When a sensor network is deployed in the furniture of a house, it is really important to know the behaviour of materials with respect to signals at 2.4 Ghz. There are materials that introduce greater amounts of losses, and this leads to a reduction of the communication link quality. This is directly related to the existence of a higher transmission error rate, which results in the need to forward the same information repeatedly. Definitely this implies higher power consumption, and the battery life decreases.

In conclusion, this work has presented a comparison between different materials that are commonly used in furniture manufacturing, and their influence on the quality of the established link between sensor and coordinator node.

The complexity of the evaluation, *i.e.*, the parameters involved for the performance of the communication link have been gradually increased, and a study was performed in a real environment of quality of link when furniture is placed between sensor and coordinator node. The obtained results confirmed the previous analysis.

Finally, we have presented the developed software tool that integrates all the gathered knowledge about the materials and communications. This tool targets two different types of users. Firstly, furniture designers could use the tool to compare the signal attenuation of different materials. Secondly, network designers of even final users could use this tool as a guide for the WSN deployment. The software tool is designed with many available options, and will return useful and practical information in an easy way.

Ongoing work is focused on the integration of sensors in furniture, in mechanical aspects as well as in communication aspects between devices, since the integration of devices which allow monitoring some user parameters (ECG, heart rate, blood pressure…) will lead to a significant increase in the importance of this type of WSN integrated in furniture at home, that certainly will be linked to a significant improvement of the quality of people's life and will serve as an impulse to the furniture industry.

This will be also evaluated the integration of the WSN's communications into IPv6 through protocols such as 6LoWPAN and GLoWBAL IPv6 [58], in conjunction with protocols such as CoAP, in order to reach a solution based on the Internet of Things and the Web of Things for sensors in furniture [59].

## Figures and Tables

**Figure 26.**
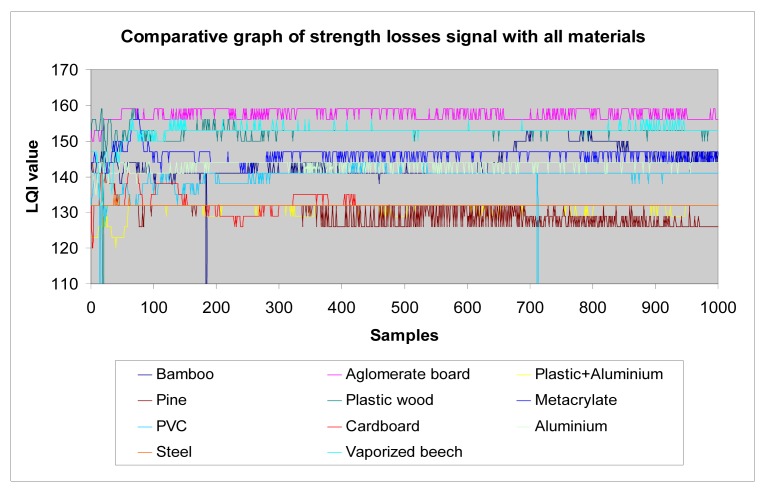
Measurements with all materials.

**Table 1.** Considered wood types.

**WOODS**	
**Type**	**Thickness (mm)**
Vaporized beech	35.51
Pine	25.55
Bamboo	20.88
Agglomerate board	16.33
Plastic wood	9.9

**Table 2.** Wood classification depending on the losses.

**Wood type**	**Thickness (mm)**	
Agglomerated board	16.33	**Lower losses**
Vaporized beech	35.51	
Plastic Wood	9.9	
Bamboo	20.88	
Pine	25.51	**Greater losses**

**Table 3.** Plastics considered.

**PLASTIC MATERIALS**	
**Type**	**Thickness (mm)**
Plastic wood	9.9
PVC	3.3
Methacrylate	3.23
Plastic + Aluminium	2

**Table 4.** Plastics classification depending on losses.

**Material**	**Thickness (mm)**	
	
Plastic wood	9.9	Lower losses
		
Methacrylate	3.23	
PVC	3.3	
		
Plastic + aluminium	2	Greater losses

**Table 5.** Materials classification depending on losses.

**Material**	**Thickness (mm)**	
Agglomerate board	16.33	**Lower losses**
Plastic Wood	9.9	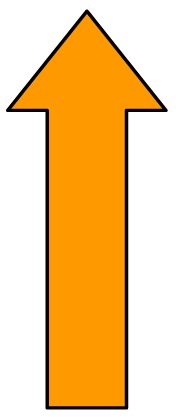
Metacrylate	3.23	
PVC	3.3	
Bamboo	20.88	
Vaporized beech	35.51	
Pine	25.51	
Cardboard	16.2	
Plastic + Aluminium	2	
Aluminium	10.39	
Steel	8.17	**Greater losses**
